# Identification of the lncRNA, AK156230, as a novel regulator of cellular senescence in mouse embryonic fibroblasts

**DOI:** 10.18632/oncotarget.10170

**Published:** 2016-06-19

**Authors:** Yu-ning Chen, Meng-yun Cai, Shun Xu, Mei Meng, Xingcong Ren, Jay W. Yang, Yu-qi Dong, Xinguang Liu, Jin-Ming Yang, Xing-dong Xiong

**Affiliations:** ^1^ Institute of Aging Research, Guangdong Medical University, Dongguan, Guangdong, P.R.China; ^2^ Key Laboratory for Medical Molecular Diagnostics of Guangdong Province, Dongguan, P.R.China; ^3^ Institute of Biochemistry & Molecular Biology, Guangdong Medical University, Zhanjiang, P.R.China; ^4^ Department of Pharmacology and The Penn State Hershey Cancer Institute, The Pennsylvania State University College of Medicine and Milton S. Hershey Medical Center, Hershey, PA, USA

**Keywords:** lncRNA, AK156230, cellular senescence, mouse embryonic fibroblasts, autophagy, Gerotarget

## Abstract

Long noncoding RNAs (lncRNAs) have gained extensive attentions in recent years because of their potential importance in a variety of biological and pathological processes. In this study, we sought to explore the role of lncRNAs in cellular senescence. Here, we report that the lncRNA AK156230 was downregulated during replicative senescence in mouse embryonic fibroblasts (MEFs), and knockdown of AK156230 promotes a robust senescence phenotype, including increase in the numbers of the senescence-associated β-galactosidase-positive cells, decrease of cell proliferation, accumulation of cells in the G2/M phase and reduction of autophagic activity. The cells with knockdown AK156230 expression also exhibited increased levels of p21, p53 and phosphorylated p53, and a decreased activity of CDK1. Moreover, rapamycin-induced autophagy offered cytoprotective effect and rescued cellular senescence in AK156230 knockdown cells. Gene expression profile showed that the dysregulation of autophagy and cell cycle genes contributed to the induction of cellular senescence after AK1561230 silencing. Taken together, these results suggest that downregulation of AK156230 is involved in the induction of cellular senescence through its roles in autophagy and cell cycle progression. Our study identifies AK156230 as a critical lncRNA that has a role in regulating cellular senescence in MEFs.

## INTRODUCTION

Replicative senescence (RS) is a cellular state of irreversible growth arrest and loss of replicative capacity [1], and can be triggered by a variety of factors such as DNA damage, telomere shortening, oncogenic activation, or oxidative stresses [2, 3]. Apart from growth arrest, senescent cells often display various characteristic phenotypes including distinct flattened, enlarged, and irregular morphology, increased activity of lysosomal β-galactosidase, robust secretion of inflammatory cytokines [4-7]. Despite diverse stress responses bring about senescent phenotype, the p53/p21 and p16/Rb pathways are the major regulators of cellular senescence in different cell types [4, 8]. Cellular senescence is also thought to contribute to tumor suppression or progression and organismal ageing. Besides, senescence has been implicated as a detrimental factor for age-related diseases such as cardiovascular disease, diabetes, neurodegenerative disease, cancer and many other diseases [9-12].

Recently, there has been an increasing appreciation of the roles of noncoding RNAs (ncRNAs) in regulating gene expression during cellular senescence [5, 13, 14]. This large class of noncoding RNAs comprise small transcripts such as siRNAs and miRNAs, and a vast majority of long noncoding RNAs (lncRNAs) [15]. LncRNAs are currently defined as transcripts of greater than 200 nucleotides that lack the protein-coding capacity [16]. Based on their transcriptional position and direction, lncRNAs can be divided into at least five categories, including sense, antisense, intergenic, intronic, and divergent lncRNAs [17]. LncRNAs have emerged as key gene regulators at multiple levels, including epigenetic chromatin modification, transcription, post-transcription, and translation, although few have been fully functionally characterized [18-20]. Recently, increasing numbers of lncRNAs have been reported to be involved in cellular processes including proliferation, differentiation, survival or apoptosis, and a variety of human diseases such as neurodegeneration, cardiovascular disease, and cancer [21-25].

In the current study, we have identified a number of lncRNAs whose expressions were altered during RS in mouse embryonic fibroblasts (MEFs). Among these lncRNAs, AK156230 was most significantly downregulated during RS, and knockdown of its expression markedly promoted the senescence-related phenotypes, including distinct flattened and enlarged morphology, reduced cell proliferation and autophagy, increased senescence-associated β-galactosidase (SA-β-gal) activity, and growth arrest. Furthermore, rapamycin-induced autophagy can be beneficial against cellular senescence induced by AK156230 silencing in MEFs. Our study suggests that AK156230 promote autophagy and delay cellular senescence in MEFs.

## RESULTS

### Differential expression of lncRNAs in senescent and young MEFs

**Figure 1:**
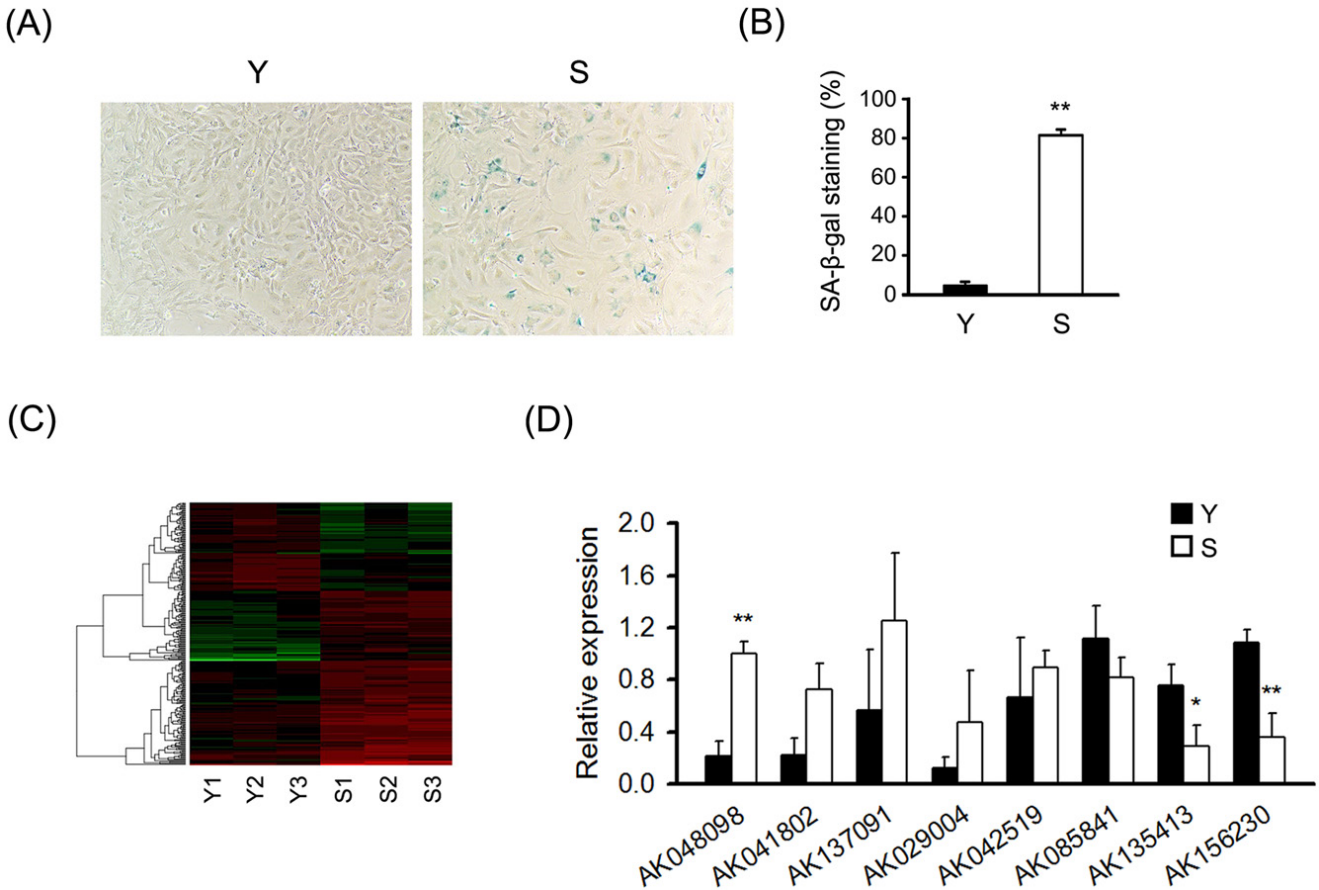
Differential expression of lncRNAs in senescent and young MEFs **A.** Micrographs to visualize senescence-associated β-galactosidase activity in senescent (S) and young (Y) MEFs. “Senescent” indicated the cells at the ninth passages; “Young” indicated the cells at the second passages. **B.** Percentage of the indicated cells positive for SA-β-gal activity was shown. Cells were quantitated by randomly choosing at least four independent fields. **C.** LncRNA expression profile of MEFs was analyzed by lncRNA microarray, heatmaps were generated from the hierarchical cluster analysis to show a distinguishable lncRNA expression profile among samples (*n* = 3). The color “Red” indicates high relative expression, and “green” indicates low relative expression. **D.** RT-qPCR analysis validation of eight differentially expressed lncRNAs in MEFs under replicative senescence (*n* = 4). β-actin was used as a loading control. All experiments represent the mean ± SEM from at least there independent experiments. Student's *t*-test, *P < 0.05, **P < 0.01.

To identify senescence-related lncRNAs, we used primary MEFs as model system. Upon senescence, cells displayed a characteristically enlarged and flattened morphology and elevated perinuclear activity of SA-β-gal, a distinguishing marker of senescent cells. Senescent MEFs showed senescent phenotypes that distinguished them from the young cells (Figure 1A and 1B). To reveal the potential role of lncRNAs in cellular senescence, we used microarray to detect differentially expressed lncRNAs and mRNAs in senescent and young MEFs. Hierarchical clustering analysis was used to gain a systematic comparison of lncRNAs and mRNAs expression between senescent and young MEFs (Figure 1C and Figure S1). The data of microarray were filtered by using the volcano plot to dispaly the differentially expressed lncRNAs and mRNAs between senescent and young MEFs (Figure S2). Our data indicated that 289 lncRNAs and 301 mRNAs were differentially expressed (fold change ≥ 2.0, *P* < 0.05) between senescent and young MEFs (Tables S1-S4; Table S6).

To validate the results of microarray analysis, we used real-time quantitative PCR (RT-qPCR) analysis on eight differentially expressed lncRNAs with fold-changes greater than 2.5. RT-qPCR experiments showed that two lncRNAs including AK156230 and AK135413 were uniformly downregulated by two-fold or greater in senescent compared with young cells. By contrast, AK048098 was remarkably upregulated in senescent relative to young cells (Figure 1D). These results suggest that AK156230, AK135413 and AK048098 are involved in MEFs senescence.

### Induction of cellular senescence phenotypes by knockdown of AK156230 in MEFs

**Figure 2:**
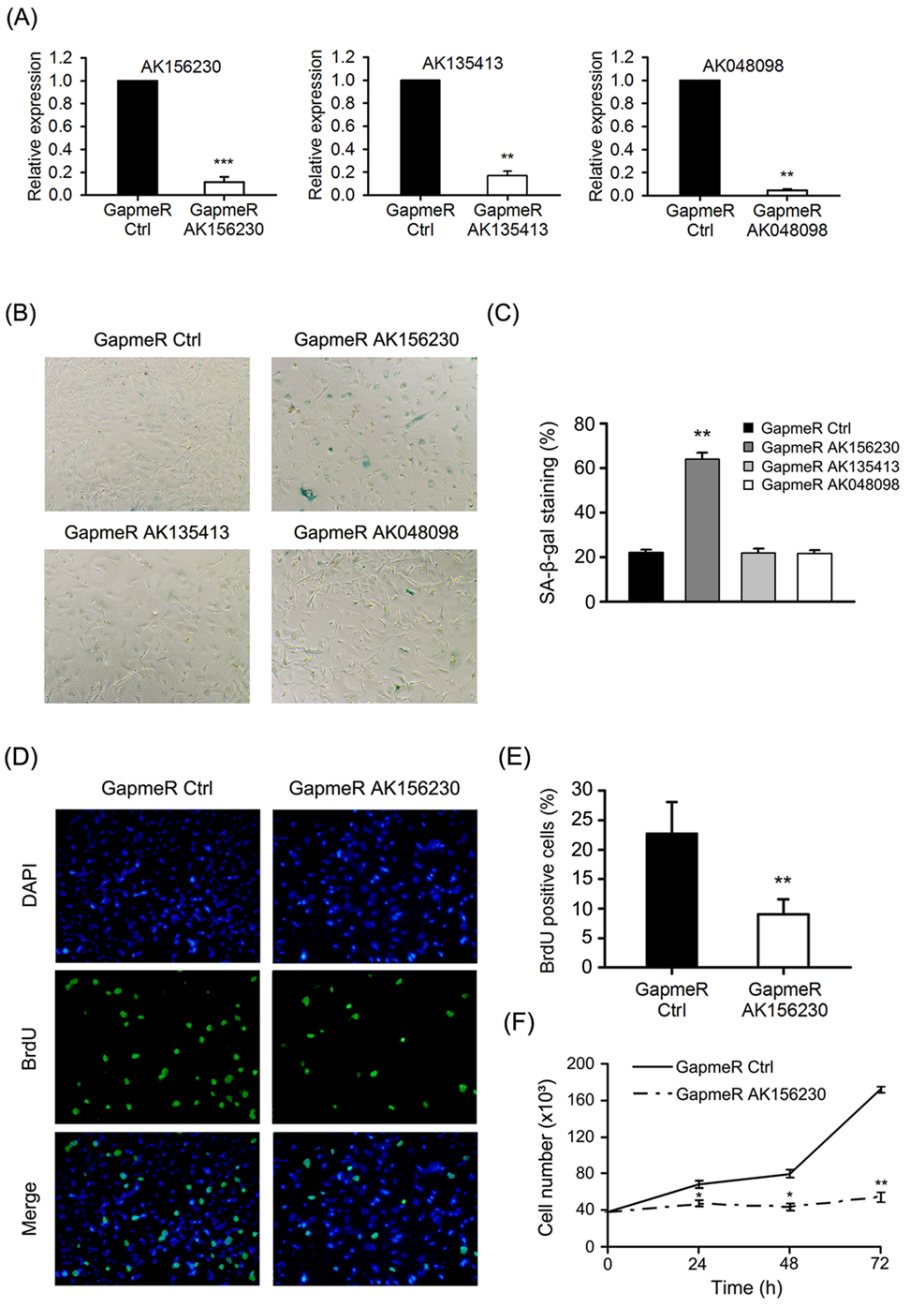
Effects of AK156230 on senescent phenotypes in MEFs **A.** Relative AK156230, AK135413 or AK048098 RNA levels in young MEFs transfected with the indicated GapmeRs for 48h analyzed by RT-qPCR. **B.** Representative images of the SA-β-gal activity staining of cells under the indicated conditions 72h post transfection. **C.** Percentage of the indicated cells positive for SA-β-gal activity was shown. Cells were quantitated by randomly choosing at least four independent fields. **D.** Proliferation of cells was measured by BrdU incorporation assay. Representative images of indicated cells stained for DAPI and BrdU by immunofluorescence were shown. MEFs at passage 2 were transfected with the indicated GapmeRs for 48h. **E.** Percentage of the indicated cells positive for BrdU was shown. Cells were quantitated by randomly choosing at least four independent fields. **F.** Growth curves of MEFs transfected with the indicated GapmeRs at the designated time points post transfection. All experiments are represented as mean ± SEM from at least there independent experiments. Student's *t*-test, *P < 0.05, **P < 0.01.

To explore the function of AK156230, AK135413 or AK048098 on cellular senescence in MEFs, we inhibited the expression of these lncRNAs by locked nucleic acid (LNA) longRNA GapmeRs (Sequences in Table S8). GapmeR induces degradation of the targeted lncRNA through an RNase H-dependent mechanism [26]. Importantly, GapmeRs treatment resulted in a reduction of total AK156230, AK135413 or AK048098 levels when compared with control, where we obtained a knockdown efficiency of 80% to 90% (Figure 2A). In young MEFs, silencing of AK156230 resulted in enlarged, flattened, and irregular morphology, with an increased SA-β-gal staining activity similar to senescent cells, while silencing AK048098 or AK135413, cells did not display a similar senescent phenotype (Figure 2B and 2C).

Cessation of cell proliferation is a hallmark of cellular senescence, thus we tested whether cell proliferation was altered when expression of AK156230 was silenced. As senescent cells are decreased in DNA synthesis, we performed immunostaining assays of 5-bromo-2′-deoxyuridine (BrdU) incorporation in MEFs. We observed that silencing of AK156230 expression resulted in the decrease of BrdU incorporation (Figure 2D). As compared with control MEFs, AK156230-knockdown cells showed a significantly lower percentage of BrdU-positive cells (Figure 2E). Subsequent analysis of growth curve revealed a remarkable reduction in cell number in MEFs with silencing of AK156230 expression (Figure 2F). The significantly diminished cell proliferation was in consistent with their premature senescence phenotypes in MEFs with knockdown of AK156230. Taken together, these results showed that silencing of AK156230 dramatically promoted cellular senescence and inhibited cell proliferation in MEFs.

### Induction of G2/M arrest and autophagy inhibition by knockdown of AK156230 in MEFs

**Figure 3:**
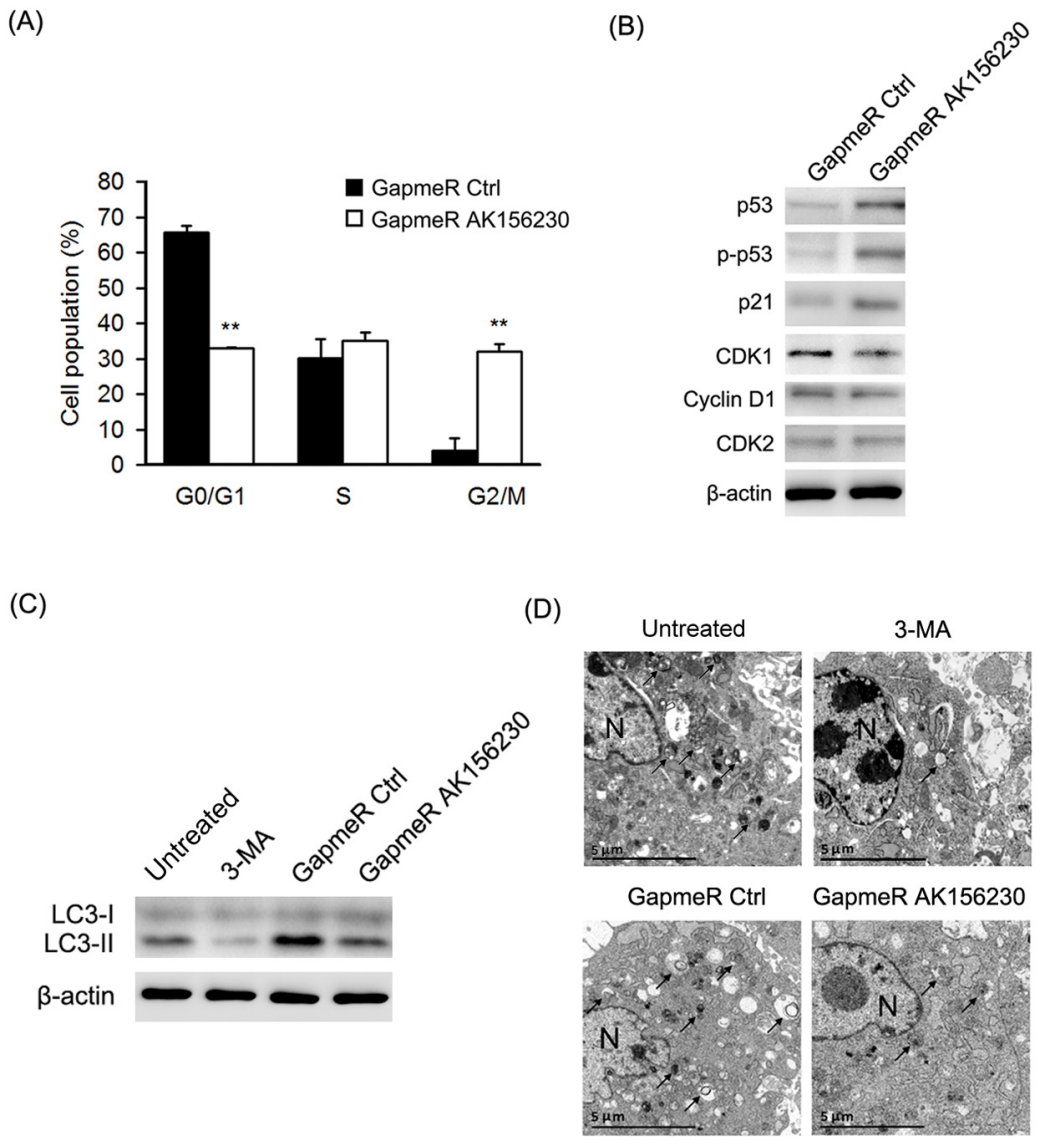
Effects of AK156230 on cell cycle progression and autophagy in MEFs **A.** Cell cycle analysis was performed at 48h after transfection in passage 2 cells transfected with the indicated GapmeRs. The percentage of G0/G1, S, and G2/M are demonstrated as shown. **B.** Western blot analysis of p53, phosphorylated p53, p21, CDK1, CDK2, and Cyclin D1 from MEFs transfected with the indicated GapmeRs for 72h. **C.** MEFs at passage 2 were transfected with the indicated GapmeRs for 48h, or treated with 3-MA (10mM) for 4h. Western blotting were performed to detect the levels of non-lipidated LC3 (LC3-I) and its lipidated variant (LC3-II). **D.** Transmission electron microscopy images of MEFs at passage 2 transfected with the indicated GapmeRs for 48h, or treated with 3-MA (10mM) for 4h. Black arrowheads indicate representative autophagosomes or autophagolysosomes, and the nucleus is denoted by *N*. These sections were examined at 120kV with a JEOL JEM-1400 transmission electron microscope. β-Actin was used as the loading control. All experiments are represented as mean ± SEM from at least there independent experiments. Student's *t*-test, **P < 0.01.

To determine whether senescence-related phenotypes seen in AK156230 knockdown cells were associated with alteration of cells cycle progression, we performed flow cytometric analysis. As shown in Figure 3A, knockdown of AK156230 expression caused a significant arrest of cell cycle progression, as evidenced by a significant increase in G2/M fraction and a decrease in G0/G1 cells, suggesting that the growth suppressive effect of AK156230 knockdown was associated with G2/M arrest. In order to explore the possible molecular mechanisms involved, we examined the expression of several key cell cycle regulators and senescence-associated genes by western blotting. Knockdown of AK156230 was accompanied by a significantly increase in p53 and phosphorylated p53 (Ser15) and p21, and a decrease in the protein levels of CDK1 (Figure 3B). These results suggest that p53 and p21 are involved in regulating cellular senescence induced by AK156230 knockdown in MEFs.

To determine whether AK156230 plays a role in autophagy, we compared the formation of LC3-II, a specific marker for autophagy, in MEFs with or without silencing of AK156230 expression. As shown in Figure 3C, silencing of AK156230 expression decreased the appearance of LC3-II in MEFs in comparison with cells treated with GapmeR control. To confirm this result, we used the autophagy inhibitor 3-methyladenine (3-MA) as a positive control, which inhibits early stages of autophagosome formation [27]. As shown in Figure 3C, the 3-MA-treated cells showed decreased conversion of LC3-I to LC3-II. Similarly, autophagic flux was assessed based on the accumulation of autophagic substrate p62 [28]. Silencing of AK156230 also significantly enhanced the abundance of p62, which was indicative of reduction in autophagy (Figure S3A).

Autophagy is a conserved catabolic process involving cytoplasmic degradation through the formation of double-membraned vesicles for recycling of the resulting basic components as metabolic precursors [29, 30]. To confirm the role of AK156230 in autophagy, we further measured autophagosome formation using transmission electron microscopy (TEM). We found that autophagosome, as evidenced by double membrane vacuoles, were prominent and easily identifiable in the cytoplasm of untreated MEFs. In contrast, very few autophagosomes were observed in AK156230-knockdown MEFs and 3-MA-treated cells (Figure 3D). These findings suggested that silencing of AK156230 exerted a negative effect on autophagy.

### Activation of autophagy improves cellular senescence induced by AK156230 knockdown

**Figure 4:**
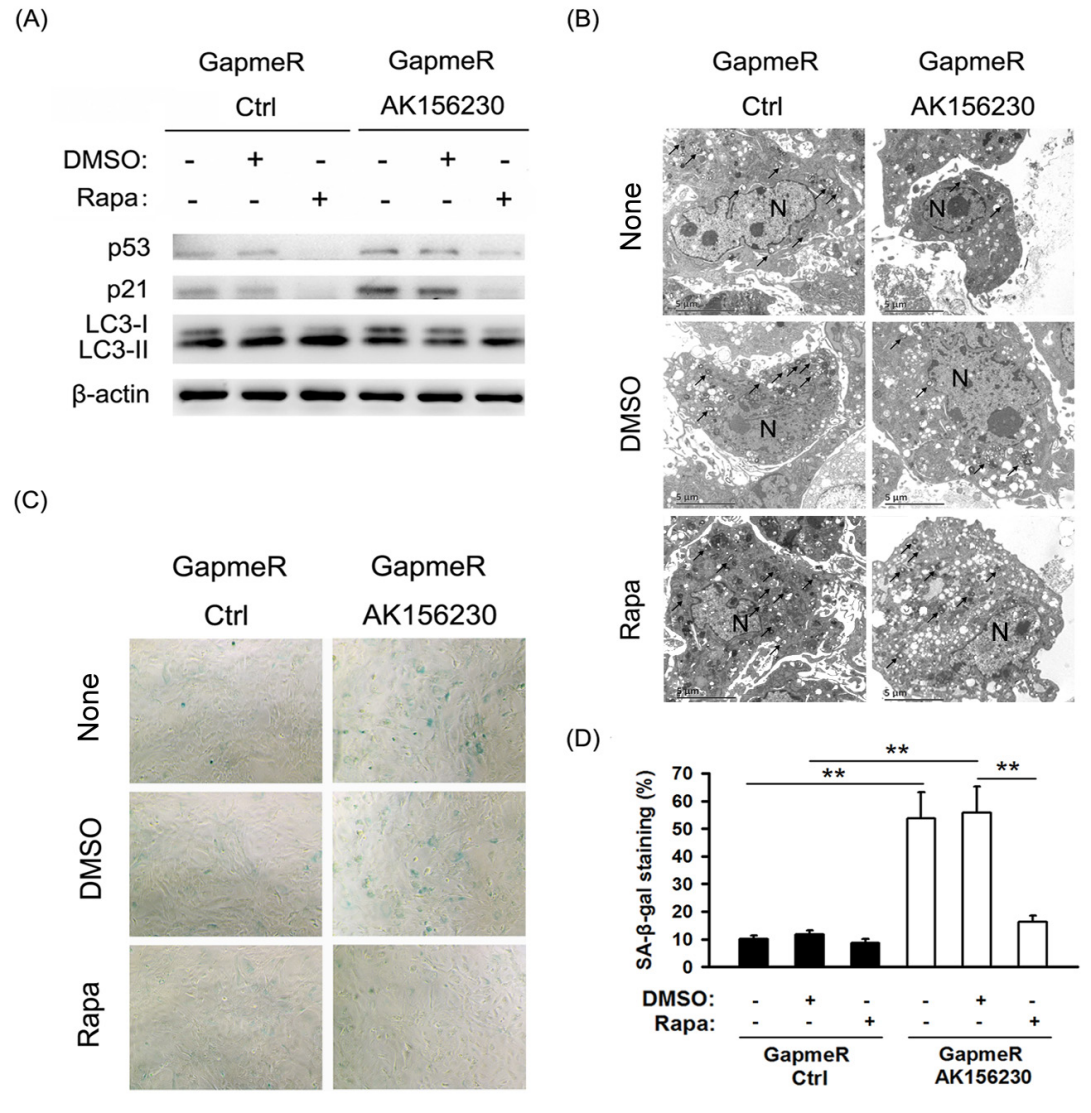
Rapamycin enhances autophagosome formation and rescues cellular senescence induced by AK156230 knockdown in MEFs **A.** Western blotting for LC3, p53 and p21 using lysates from the indicated cells. β-Actin was used as the loading control. **B.** Transmission electron microscopy images of MEFs treated with 2.5μM rapamycin or DMSO for an additional 48h after transfection with control GapmeR control or GapmeR AK156230 for 24h. Black arrowheads indicate representative autophagosomes or autophagolysosomes, and the nucleus is denoted by *N*. These sections were examined at 120kV with a JEOL JEM-1400 transmission electron microscope. **C.** Representative images of the SA-β-gal activity staining for the indicated cells. **D.** Percentage of the indicated cells positive for SA-β-gal activity was shown. Cells were quantitated by randomly choosing at least four independent fields. Student's *t*-test, **P < 0.01.

We next investigated whether autophagy induction by rapamycin can be beneficial against cellular senescence induced by AK156230 knockdown. As shown in Figure 4A, rapamycin treatment could rescue the AK156230 knockdown-induced autophagy attenuation, and concomitantly reversed AK156230 knockdown-mediated p53 and p21 expression. However, when AK156230 knockdown cells were treated with rapamycin, the number of autophagosomes increased significantly, as determined by TEM (Figure 4B). Notably, as compared with AK156230 knockdown cells, a significantly decrease in MEFs senescence was observed in those cells in the presence of rapamycin, as measured by means of SA-β-gal staining (Figure 4C and 4D). These data suggest that autophagy induction by rapamycin offers cytoprotective effect and rescues cellular senescence in AK156230 knockdown cells.

### AK156230 knockdown is associated with a senescence-related signature

**Figure 5:**
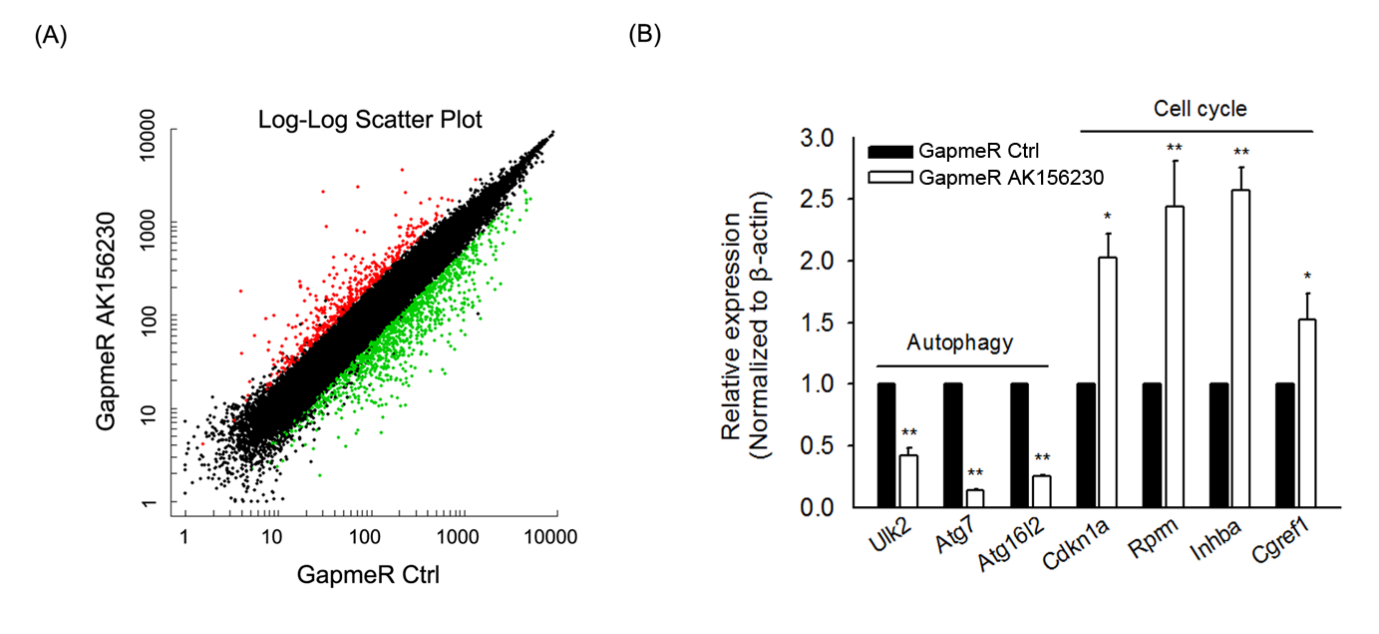
Gene expression signature induced by AK156230 silencing **A.** Scatter plot of differentially expressed genes from MEFs transfection with GapmeR control or GapmeR AK156230. **B.** The expression of autophagy and cell cycle-related genes in MEFs at 48h after transfection with GapmeR control or GapmeR AK156230 was measured by RT-qPCR analysis. All experiments are represented as mean ± SEM from at least there independent experiments. Student's t-test, **P < 0.01.

In order to determine which function of AK156230 might be involved in its role in senescence, we performed mRNA microarray to screen the differentially expressed mRNAs after AK156230 knockdown. The microarray data showed that 1633 genes were differentially expressed (Fold change ≥ 2.0; Tables S5 and S9). To further characterize the genes that are affected by AK156230 expression, we searched for enrichment of genes annotated to shared biological processes among genes that were up- or down-regulated following transfection with GapmeR AK156230 (Table S10). Such processes included several genes related to autophagy and cell cycle progression (Table S10). Pathway analysis showed that several gene categories including “p53 signaling pathway” and “cell cycle” might play important roles in the cellular senesecence, and the gene categories “regulation of autophagy” and “mTOR signaling pathway” were involved in the autophagy process (Table S11). We further tested a subset of these genes associated with autophagy and cell cycle by qPCR. As shown in Figure 5, knockdown of AK156230 was accompanied by a significant decrease in autophagy-related genes including Ulk2, Atg7 and Atg16l2, and an increase in anti-proliferative genes including Cdkn1a, Rprm, Inhba and Cgref1, showing a strong consistency between the RT-qPCR results and the microarray data.

## DISCUSSION

In recent years, great efforts have been made in understanding the expression patterns and functions of lncRNA, and the importance of these non-coding RNAs has been increasingly appreciated [31-33]; yet, so far only a small portion of this class of non protein-coding transcripts are known to be involved in regulating cellular senescence, and those results were obtained in different types of human tissues and cells. For example, Gorospe's group has identified a number of senescence-associated lncRNAs in human diploid fibroblasts [34]. More recently, it was reported that the lncRNAs, ASncmtRNA-2 and MIR31HG, were closely related to cellular senescence and aging process [35, 36]. As mouse is a commonly used model in aging studies including those involve testing of anti-aging approaches, here we sought to identify the lncRNAs and their roles in regulating aging in mouse. We believe that such information would be useful for better utilization of mouse models in aging studies and for better interpretation of the data obtained in mouse models.

Our microarray profiling revealed a number of lncRNAs whose abundance were altered during senescence in MEFs (Figure 1, Tables S1 and S2); however, we only identified AK156230 as a regulator of cellular senescence, as RNAi-mediated knockdown of AK156230 induced a switch of the cell phenotype to an anti-proliferative state, leading to accelerated cellular senescence, as determined by SA-β-gal staining assay (Figure 2). By contrast, we did not observe any effects on cellular senescence with other lncRNAs (e.g., AK135413 or AK048098) whose expressions were also found to be changed during senescence process, suggesting that not every lncRNA showing changes during senescence has functional roles. It is likely that the altered expressions of these lncRNAs are the consequence of cellular senescence rather than its causes. Further studies would be needed to explore the biological implications of these lncRNAs.

The impact of AK156230 in controlling cellular senescence is supported by several lines of evidence. First, we show that knockdown of AK156230 in MEFs significantly promotes cellular senescence (Figure 2C), and inhibits cell proliferation, as determined by BrdU incorporation and cell growth assay (Figure 2D-2F). Second, we demonstrate a remarkable senescence-like cell cycle arrest at G2/M phase in MEFs subjected to AK156230 knockdown (Figure 3A). Examination of the expression levels of several genes involved in senescence and cell cycle control also supports the role of AK156230 in negative regulation of replicative senescence (Figure 3B).

Attenuation of cell cycle progression is considered an important mechanism in the development of growth arrest and cellular senescence [37]. It is well known that CDK1 kinase plays a crucial role in regulation of cell cycle from G2 phase into M phase [38-42]. We show here that knockdown of AK156230 resulted in a decrease in the expression of CDK1, suggesting that downregulation of CDK1 significantly induced G2/M arrest. Additionally, in the MEFs with knockdown of AK156230 expression we observed a significant induction of p53 and p21 (Figure 3B). Therefore, these data suggest that AK156230 knockdown may block cell cycle progression in MEFs through p53/p21/CDK1 pathway.

By examining the appearance of LC3-II and autophagic vesicle and the abundance of p62, we show that knockdown of AK156230 downregulated autophagy, a cellular process critically associated with aging. However, p62 mRNA expression did not increased in AK156230-knockdown MEFs (Figure S3B), suggesting that the observed increase in p62 protein expression upon AK156230 knockdown-mediated suppression of autophagy was a post-transcriptional response rather than transcriptional regulation. In addition, as transcriptional regulators, some lncRNAs can regulate transcription positively or negatively on neighboring genes. In this study, we also screen the differentially expressed mRNAs in senescent and young MEFs (Tables S3 and S4). However, we did not observe any significant differences in neighboring genes (eg. Adam22 and Sorcin) around AK156230, which is available from GEO under accession number GSE74435.

Autophagy, a major interacellular degradation and recycling system, plays an important role in the control of cellular senescence. Increasing studies have found that restoring autophagy by rapamycin rescues cellular senescence [43-44]. Here we found that rapamycin-induced autophagy rescued cellular senescence in AK156230 knockdown cells (Figure 4), suggesting that rapamycin-induced autophagy can be beneficial against cellular senescence induced by knockdown of AK156230 in MEFs. In addition, the dysregulation of autophagy and cell cycle genes observed by gene expression profiling are consistent with autophagy suppression and anti-proliferative effect after AK1561230 silencing (Figure 5). All of these evidences indicate an inhibitory role for AK156230 in cellular senescence, but the precise mechanism by which this lncRNA delays cellular senescence and its *in vivo* effects on this cellular process and aging remain to be elucidated. Additional experiments are required to establish how AK156230 functions, and validate whether the phenotypes of AK156230 knockdown are truly linked to AK156230 loss through rescue experiment. The ideal strategy should capture in lncRNA-protein/DNA/RNA interactions, and provide comprehensive portraits of AK156230 in cellular senescence.

Taken together, we have identified AK156230 as a negative regulator of cellular senescence in MEFs. The results of this study may be informative about the lncRNA biology and potentially useful for comparative studies of cellular and molecular pathways that affect aging in mouse and other species including human.

## MATERIALS AND METHODS

### Cell cultures

Primary mouse embryonic fibroblasts (MEFs) were isolated from 13.5-day-old embryos of C57/BL6 mice and maintained in Dulbecco's modified Eagle medium (DMEM, Gibco, Grand Island, NY, USA) supplemented with 10% fetal bovine serum (FBS, Gibco, Grand Island, NY, USA) in a humidified incubator with 5% CO_2_ at 37°C. Serial passage was performed when the cells reached 80% conﬂuence.

### Microarray analysis

The microarray with coverage of 41597 mouse lncRNAs and 35291 mouse mRNAs were synthesized by Ribobio applying the Combimatrix platform to detect differentially expressed lncRNAs and mRNAs in senescent and young MEFs. All procedures were carried out according to manufacturer's protocol. The microarray hybridization and bioinformatic analysis was performed using the RiboArray platform (Ribobio, Guangzhou, China). Briefly, total RNA from senescent and young MEFs was quantified by the K5500 micro-spectrophotometer (Kaiao, Beijing, China). The quality of total RNA was assessed using the Agilent 2200-Bioanalyzer (Agilent, Santa Clara, CA, USA). Two μg of total RNA was reverse transcribed into aRNA and labeled using Amino Allyl MessageAmp II aRNA kits (Life Technologies, Carlsbad, CA, USA). The concentration and specific activity of the labeled aRNAs were measured using a K5500 micro-spectrophotometer. For hybridization, microarrys with labeled samples were hybridized at 40°C for 16h in the hybridization chamber and washed using buffers recommended in the manufacturer's protocol. Slides were scanned using a Genepix 4000B laser scanner (Molecular Devices, Sunnyvale, CA, USA) and images were analyzed using Genepix Pro 7.0 software (Molecular Devices, Sunnyvale, CA, USA). Differentially expressed lncRNAs were obtained using linear models for microarray data analysis, and selected based on *P* < 0.05, fold changes ≥ 2.0. The microarray data from this study have been submitted to the NCBI Gene Expression Omnibus (GEO) under accession number GSE74435 (http://www.ncbi.nlm.nih.gov/geo/query/acc.cgi?acc=GSE74435).

For gene expression profiling after AK156230 silencing, cells were transfected with control GapmeR or GapmeR directed against AK156230 and were subjected to extraction of total RNA. Briefly, RNA amplification and labeling were done according to the manufacturer's protocol (Affymetrix, Santa Clara, CA). Fragmented cRNA was individually hybridized with the GeneChip Porcine Genome Array (Affymetrix, Santa Clara, CA), which contained 45,038 probes. For hybridization, microarrys with labeled samples were hybridized at 45°C for 16h according to manufacturer's instructions. The GeneChip arrays were scanned immediately after washing and staining using a GeneChip Scanner 3000 (Affymetrix, Santa Clara, CA) and hybridization data were analyzed using GeneChip Operating Software (Affymetrix, Santa Clara, CA). In a comparison analysis, a two class unpaired method in the Significant Analysis of Microarray software (SAM) was applied to identify significantly differentially expressed genes between AK156230 knockdown and control groups. The microarray data from this study have been submitted to the NCBI Gene Expression Omnibus (GEO) under accession number GSE81829 (http://www.ncbi.nlm.nih.gov/geo/query/acc.cgi?acc=GSE81829).

### Transfections

The day before transfection, MEFs were plated in growth medium at a density of 60% to 70% confluency. Transfection of LNA longRNA GapmeR (Exiqon, Vedbaek, Denmark) targeting AK156230 or AK135413 or AK048098 were performed using Lipofectamine RNAiMax (Life Technologies, Carlsbad, CA, USA) according to the manufacturer's instructions. The non-targeting scramble LNA GapmeR was used as a control. Fifty nM of LNA GapmeR were used for each transfection.

### RNA extraction and real-time quantitative RT-PCR analysis

Total RNAs from cultured cells were extracted using Trizol reagent (Invitrogen, Carlsbad, CA, USA), and then were reverse transcribed into cDNA using a PrimeScript^TM^ RT reagent Kit with gDNA Eraser (Perfect Real Time) (Takara, Dalian, China) according to the manufacturer's instructions. Real-time PCR was performed in an ABI 7500 Real-Time PCR System (Applied Biosystems, Austin, CA, USA) with SYBR^®^ Select Master Mix (Applied Biosystems, Foster City, CA, USA). The amplification condition was as follows: 50°C for 2 minutes, 95°C for 2 minutes, then 40 cycles at 95°C for 15 seconds, 60°C for 1 minute. Dissociation curves were obtained to confirm the specificity of the amplified DNA. The primers sequences are listed in Tables S7 and S12. β-actin was used as the internal control. The relative expression of lncRNAs was calculated by the 2^-ΔΔCt^ method after normalization against the expression of β-actin.

### Senescence associated β-galactosidase staining

Cells were stained using the Senescence Cells Histochemical Staining Kit (Sigma Aldrich, St Louis, MO, USA) following the manufacturer's instructions. In brief, cells were washed once in 1×PBS, fixed for 7 min in 1×fixative solution, and washed twice in 1×PBS, follow by stained with fresh SA-β-gal staining solution at 37°C for 48h. The percentage of positively stained cell (blue cells) *versus* total cells was counted by randomly choosing six microscopic fields. The images were captured at 100× magnification using a Nikon Eclipse TS100 microscope (Nikon Corporation, Tokyo, Japan).

### BrdU incorporation

Cell proliferation was analyzed by BrdU incorporation assay. Briefly, cells were labeled with 40μM BrdU (Sigma Aldrich, St Louis, MO, USA) for 1h, fixed with 4% paraformaldehyde, and immunostained with a mouse anti-BrdU antibody (Cell Signaling Technology, Danvers, MA, USA) followed by staining with an anti-mouse secondary antibody conjugated to Alexa Fluor 488 (Cell Signaling Technology, Danvers, MA, USA) and counter-stained with 4′,6-diamidino-2-phenylindole dihydrochloride (DAPI, Molecular Probes, Eugene, OR, USA). BrdU-positive cells were visualized and images were captured at 100× magnification using a fluorescent microscope (Olympus, Tokyo, Japan) and presented as the percentage of BrdU-positive nuclei over total number of nuclei counted. Cells were quantitated by randomly choosing at least four independent fields. At least 600 nuclei were counted.

### Cell growth

Cells were plated into 24-well plates at an initial concentration of 3×10^5^ cells per well 24h prior to transfection. After 24h, the cellular concentrations were determined using the Cellometer (Nexcelom Bioscience, Lawrence, MA, USA). Then, cells were transfected with LNA GapmeR AK156230 or a GapmeR Control. On each subsequent day, cells (triplicates) were trypsinized, resuspended in 10% FBS, and counted with the Cellometer. This procedure was performed for three consecutive days at which point cells began to approach confluency.

### Transmission electron microscopy

Cells were fixed with 2.5% glutaraldehyde in 0.1 mol/L sodium phosphate buffer (pH 7.4), washed in the same buffer five times, post-fixed in 1% osmium tetroxide in 0.1 mol/L sodium phosphate buffer for 1 h, and then dehydrated and embedded in Epon SPI812. Ultrathin sections were stained with uranyl acetate and lead citrate. Sections were examined for autophagosomes at 120kV with a JEOL JEM-1400 transmission electron microscope (JEOL, Tokyo, Japan).

### Autophagy modifying drugs

3-Metyladenine (3-MA) and rapamycin (Rapa) were obtained from Sigma-Aldrich (St. Louis, MO). For 3-MA treatment studies, cells were seeded and let adhere on sterile plastic dishes for 24h prior to start any treatment. After 24h, the cells were treated with 10mM 3-MA for further 4h. For rapamycin treatment studies, the cells were treated with 2.5μM rapamycin or DMSO (Invitrogen, Carlsbad, CA, USA) for further 48h after transfection of GapmeR AK156230 or GapmeR control. All experiments were carried out in triplicate.

### Cell cycle analysis

Briefly, Cells were trypsinized, washed with 1×PBS, and then fixed with 70% ethanol for overnight at 4°C. Following centrifugation, 500g for 5 min at 4°C, the fixed cells were washed with 1×PBS, and resuspended with 500ul of propidium iodide (PI) staining solution (0.2% Triton X-100, 100 μg/ml RNase, 50 μg/ml PI in 10 ml of PBS) and incubated in the dark for 30 min at room temperature. Flow cytometry analysis was performed on a BD FACSCanto II flow cytometer (BD Biosciences, San Jose, CA, USA), and the proportion of cells in the G0/G1, S, and G2/M phases of the cell cycle were modeled using Wincycle software (Phoenix Flow Systems, San Diego, CA, USA).

### Western blot analysis

Cell lysates were extracted, separated by SDS-poly-acrylamide gel electrophoresis, and transferred onto PVDF membranes (Millipore, Billerica, MA, USA). Immobilized membrane was incubated with primary antibodies and probed with the respective secondary antibodies conjugated with HRP (Bethyl Laboratories, Montgomery, TX, USA). The proteins were visualized using enhanced chemiluminescence (ECL) substrate kit (Thermo Scientific, Rockford, IL, USA). The following primary antibodies were used in this study: p21, CDK1 (Abcam, Cambridge, MA, USA); phosphorylated p53 (Ser 15), p53, CDK2, Cyclin D1, p62 (Cell Signaling Technology, Danvers, MA, USA); LC3B (NOVUS Biologicals, Littleton, CO, USA) and β-actin (Santa Cruz Biotech, Santa Cruz, CA, USA).

### Statistical analysis

All data were expressed as mean ± SD unless otherwise stated. All experiments were repeated three times, each with triplicate samples. Statistical differences were determined by the student's *t*-test, and values of *P* < 0.05 were considered statistically significant.

## SUPPLEMENTARY MATERIAL FIGURES AND TABLES













## Abbreviations

MEFs: mouse embryonic fibroblasts
SA-β-gal: senescence-associated β-galactosidase
LncRNAs: long noncoding RNAs
mtDNA: mitochondrial DNA
RS: replicative senescence
OIS: oncogene-induced senescence
TEM: transmission electron microscopy.
