# Supplementary material for: Identification of the lncRNA, AK156230, as a novel regulator of cellular senescence in mouse embryonic fibroblasts
Source: Oncotarget. 2016 Jun 19;7(33):52673–84. doi: 10.18632/oncotarget.10170 (PMC5288140; doi:10.18632/oncotarget.10170)
Supplement: Supplementary file 1 [file oncotarget-07-52673-s001.pdf]

Identification of the lncRNA, AK156230, as a novel regulator of cellular senescence in mouse embryonic fibroblasts

Supplementary Material

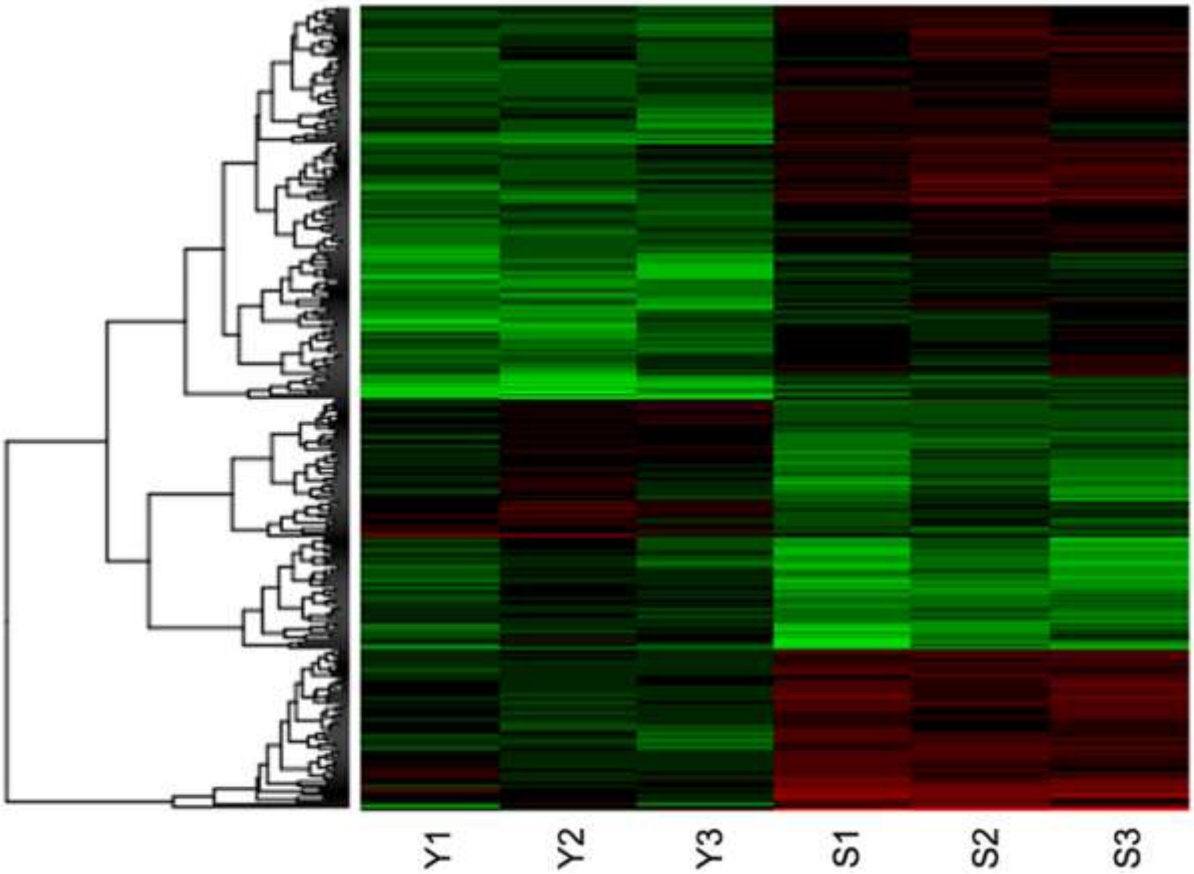

Supplementary Figure S1: mRNA expression profiles in senescent and young MEFs

(A)

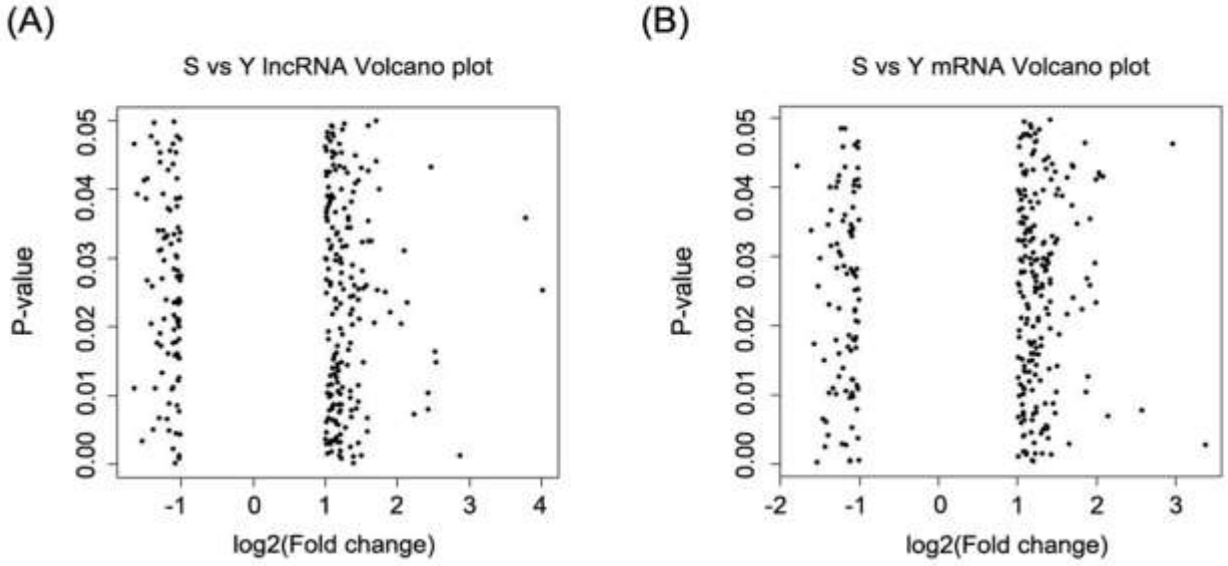

Supplementary Figure S2: Volcano plot of differentially expressed lncRNAs and mRNAs in senescent and young MEFs.

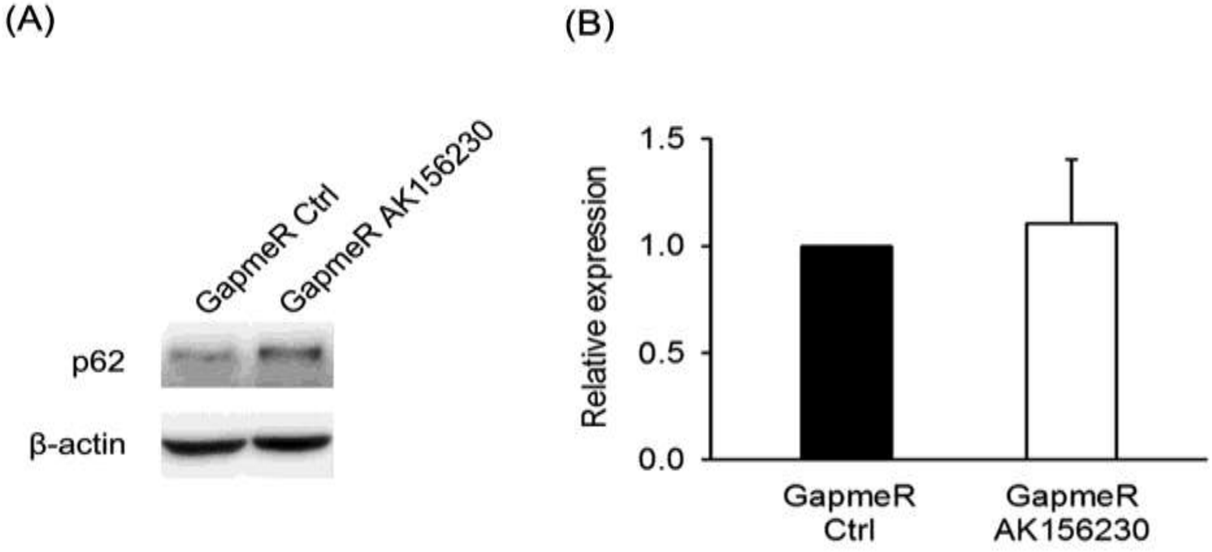

Figure S3: p62 expression after silencing of AK156230 in MEFs.

Table S6: Numbers of expressed differentially lncRNAs and mRNAs in senescent and young MEFs

	Fold change 2-3	Fold change 3-4	Fold change >4	total
**lncRNA**				
Up-regulation	168	12	12	192
Down-regulation	94	3	0	97
**mRNA**				
Up-regulation	182	18	7	207
Down-regulation	92	2	0	94

Table S7: Primers used for RT-qPCR analysis to validate lncRNAs expression

Primer	Sequence (sense)	Sequence (antisense)
AK137091	GGGTCGTTGGTTGGTTTC	TCATTAGGCGATTGGTTCAC
AK029004	TTGGGTACTTCCTCTGTGCCT	CAACGAAGCTAAGAGCTGGC
AK048098	TTCTCTGATGACTGCCGAGC	GGAACCCATACCCAACCCT
AK042519	CCTTAGGGCTGCCTGATTC	TTGGTTATAGCTGGTATGGTGC
AK041802	ACGGCATCGCATCGTATGT	TCCAGCATCCTCAGGGTCTG
AK085841	CAAGGTCCGTGGTTTGTCC	CACTTACCCATTTCTTCTGTTCC
AK135413	GCCCACTGACACCTCCTATT	ATCAGACCCTTTCTCCAGTTG
AK156230	GAGGCTTTGAAAGTATGCTGC	AAGTGGGGTGTTGAATTGGT
β-actin	AGATGACCCAGATCATGTTTGAG	AGAGCATAGCCCTCGTAGAT

Table S8: Sequences for LNA longRNA GapmeR

LNA GapmeR	Sequence
GapmeR AK156230	GGAATAAAGATGGACT
GapmeR AK135413	AACTAACAGTAATGGA
GapmeR AK048098	TTTAGCATGCTTGGTT
GapmeR Ctrl	AACACGTCTATACGC

Table S9: Numbers of differentially expressed mRNAs for AK156230 knockdown

mRNA	Fold change 2-4	Fold change 4-8	Fold change >8	total
Up-regulation	412	45	19	476
Down-regulation	900	197	60	1157

Table S10: GO term (Biological process) for the differentially expressed transcripts after AK156230 knockdown

GO.ID	Term	Count	*p*-Value	*q*-Value
GO:0007050	cell cycle arrest	16	4.493E-10	3.019E-08
GO:0008285	negative regulation of cell proliferation	31	6.512E-10	4.213E-08
GO:0016242	negative regulation of macroautophagy	6	1.204E-09	6.789E-08
GO:0000046	autophagic vacuole fusion	4	3.145E-07	9.082E-06
GO:0045786	negative regulation of progression through cell cycle	11	0.000151	0.001576
GO:0001302	replicative cell aging	2	0.010895	0.040715
GO:0048147	negative regulation of fibroblast proliferation	2	0.014300	0.050116
GO:0030308	negative regulation of cell growth	5	0.015794	0.054104
GO:0000086	G2/M transition of mitotic cell cycle	2	0.022272	0.063341
GO:0006914	autophagy	3	0.072929	0.131688

Table S11: KEGG pathway terms for the differentially expressed transcripts after AK156230 knockdown

KEGG Pathway	Count	*p*-Value	*q*-Value
p53 signaling pathway	7	1.43906E-06	5.58286E-06
Cell cycle	6	0.000366409	0.000746731
mTOR signaling pathway	5	4.7086E-05	0.000135372
Regulation of autophagy	2	0.028836139	0.029016365
Focal adhesion	27	6.5182E-26	1.04943E-23
Axon guidance	23	9.31464E-25	7.49828E-23
ECM-receptor interaction	19	3.433E-23	1.84238E-21
Cytokine-cytokine receptor interaction	26	9.76253E-22	3.92942E-20
Biosynthesis of steroids	11	5.16602E-18	1.66346E-16
Starch and sucrose metabolism	12	4.78614E-17	1.28428E-15

Table S12: Primers used for RT-qPCR analysis to validate mRNAs expression

Primer	Sequence (sense)	Sequence (antisense)
ULK2	TGGGAATGACCCTCGTGAG	AGCTACATCCATTGGTCGTTCT
ATG7	CGCCAAGATCTCCTACTCCAAT	GGCATTCACTCCGGGAAATA
ATG16L2	CGAATCAGCAATATCCGACAG	CACCAAGCCACGGCATTA
CDKN1A	AGGCACCATGTCCAATCC	AAGTCAAAGTTCCACCGTTCT
Rprm	GGTCAACAGTAGCGAGGCG	CAGCCAAGGAAGAAGATGCC
Inhba	CCAAAGTCACCATCCGTCTA	GGTACTCTTCCGAGCATCAAC
Cgref1	GGACCCTGAGGTACAACAGC	GTCATGAAGAGCAAAGAGCG
P62	TCTCTACAGATGCCAGAATCG	CTGTAGGAGCCTGGTGAGC
β-actin	AGATGACCCAGATCATGTTTGAG	AGAGCATAGCCCTCGTAGAT
